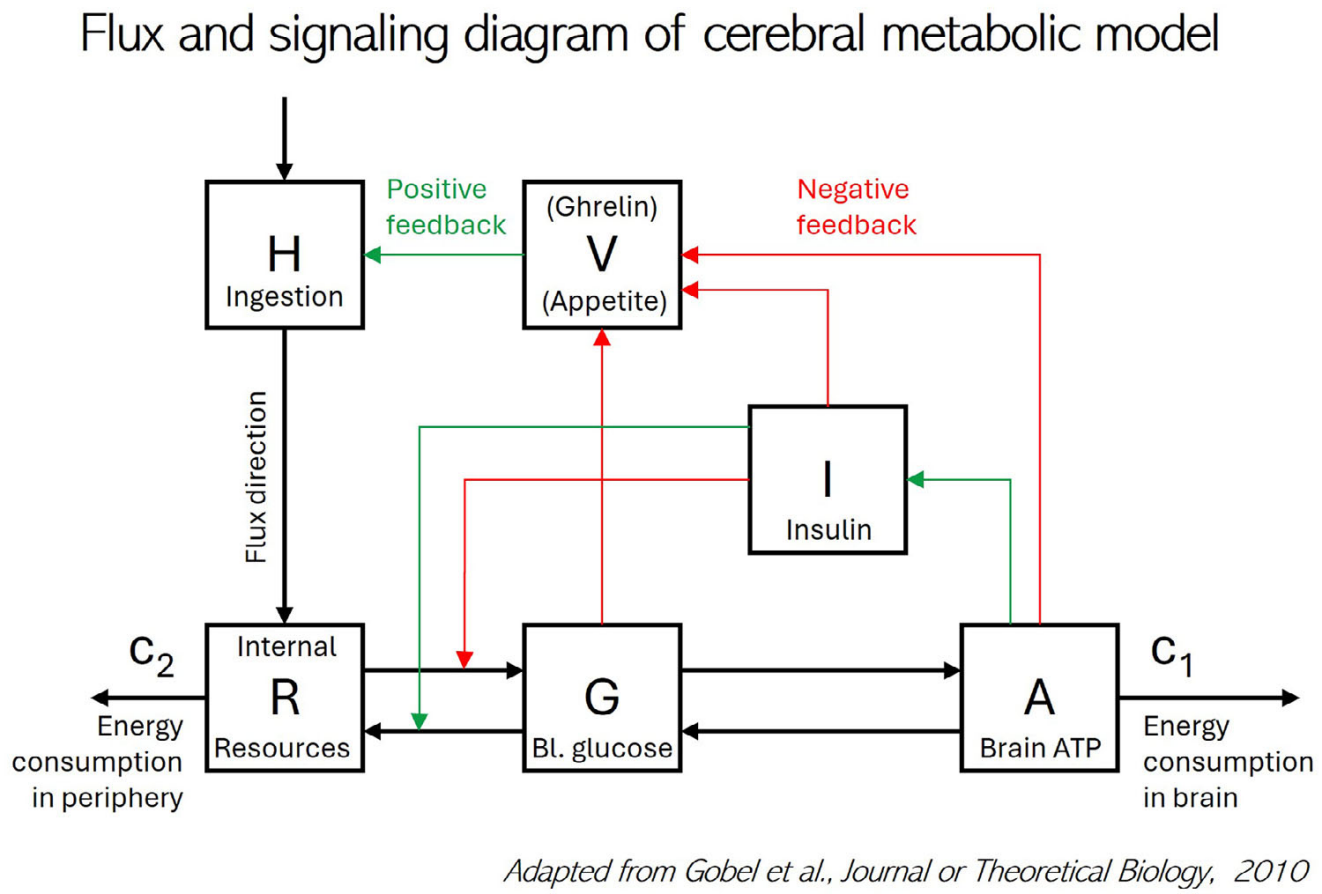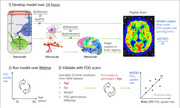# Integrating metabolism into a computational model of brain health across the human lifespan

**DOI:** 10.1002/alz70855_104136

**Published:** 2025-12-23

**Authors:** Parissa Fereydouni‐Forouzandeh, Nicolas Doyon, Simon Duchesne

**Affiliations:** ^1^ Quebec Heart and Lung Institute Research Centre, Quebec City, QC, Canada; ^2^ Laval University, Quebec City, QC, Canada; ^3^ CERVO Brain Research Centre, Quebec City, QC, Canada

## Abstract

**Background:**

There remains uncertainty on the pathogenesis of AD, with diverging unifactorial theories continuously being researched. We recently proposed a multi‐scale, multi‐factorial causal framework of brain health to bridge this theoretical gap. Unlike traditional statistical or data mining methodologies, this theoretical mathematical model is built from first principles using ordinary differential equations (ODEs). However, it did not consider the progressive downregulation of metabolic changes in AD.

**Method:**

We will extend our model with the brain‐centered metabolic model of Göbel (Göbel et al., 2010). It consists of five ODEs that account for the variations in concentrations of brain ATP, blood glucose, and plasma insulin, as energy ingested is set against stored resources. We are modifying the Göbel model by including diurnal (24 hours) constraints on ingested resources (i.e. a sleep phase); energy balance (ingestion equally or being superior to demand, leading to obesity); and inhomogeneity of consumption within brain regions, based on estimated local cell concentrations. Our model will encapsulate a 24‐hour metabolic period. Theoretical predictions will be validated against cohort data on human participants (*N* >> 3,500) including the Alzheimer's Disease NeuroImaging Initiative (Figure 1).

**Result:**

We replicated the original model, set to run over about 12 hours in the daytime. Due to inconsistencies, model parameters are being validated in the literature. ODEs are being modified to complement the model on a full 24 hour‐cycle. Concentration patterns are reviewed from the literature for diurnal profiles of glucose, insulin, and ghrelin (appetite signaling hormone).

**Conclusion:**

Having a comprehensive brain health model across the lifespan that includes metabolism would allow us to estimate the metabolic trajectory leading up to symptomatic appearance. We could then estimate the future trajectory of AD biomarkers based on a person's risk factor profile, thus providing a starting point for earlier AD diagnosis.

Reference: Göbel, B., Langemann, D., Oltmanns, K. M., & Chung, M. (2010). Compact Energy Metabolism Model: Brain Controlled Energy Supply. *Journal of Theoretical Biology, 264*(4), 1214–1224. https://doi.org/10.1016/j.jtbi.2010.02.033